# Supplementary material for: Ventral midbrain stimulation induces perceptual learning and cortical plasticity in primates
Source: Nat Commun. 2019 Aug 9;10:3591. doi: 10.1038/s41467-019-11527-9 (PMC6689065; doi:10.1038/s41467-019-11527-9)
Supplement: Supplementary file 1 — Supplementary Information [file 41467_2019_11527_MOESM1_ESM.pdf]

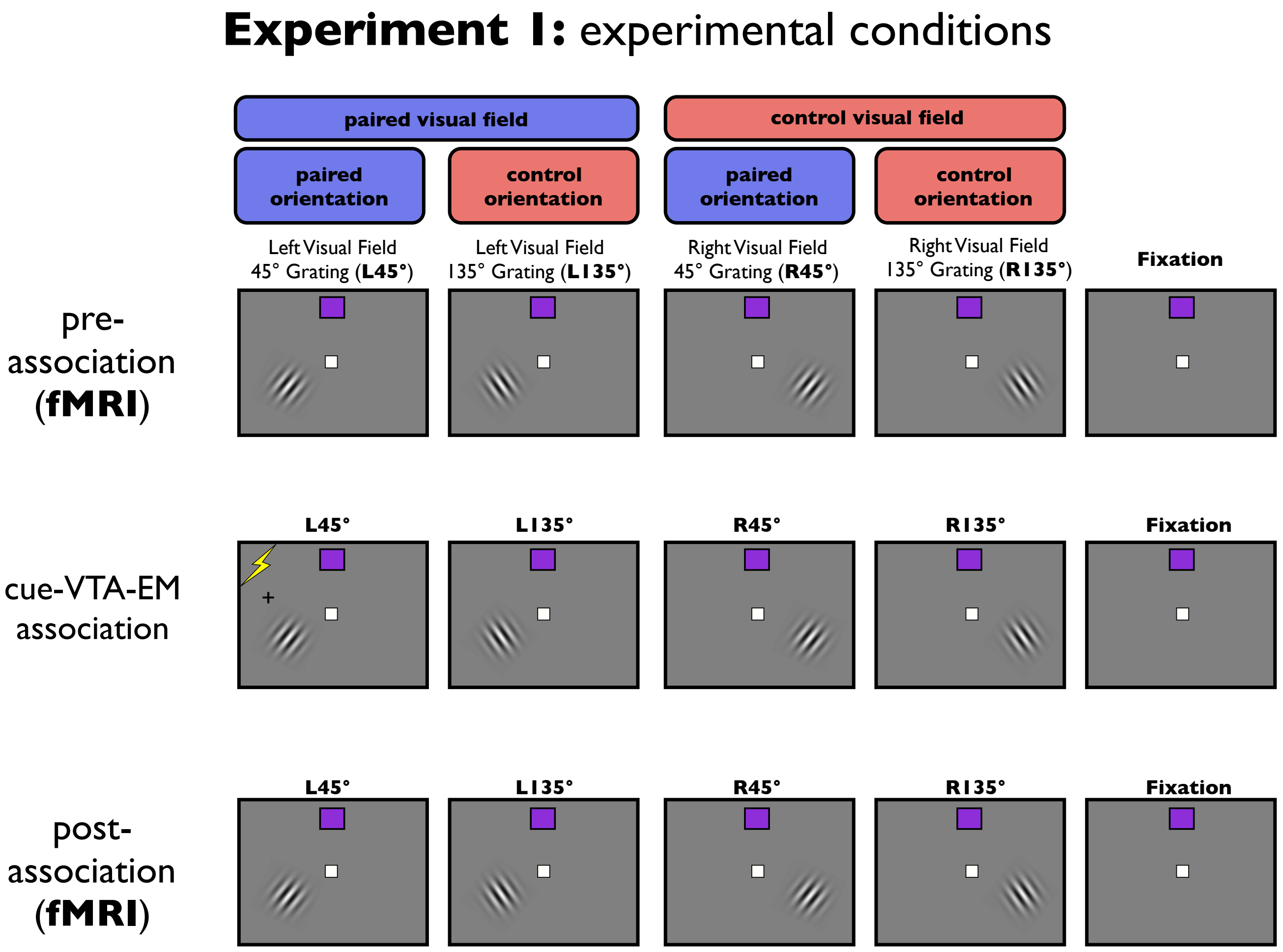

Supplementary Figure 1 | Experiment 1 - Experimental conditions. Schematic of all experimental conditions used in experiment 1 across the 3 phases of the experiment (pre-association, cue-VTA-EM association, post-association). This schematic is of an experiment in which the 45° grating presented in the LVF was associated with VTA-EM in the cue-VTA-EM association phase and therefore the L45 condition is considered the paired stimulus. The oriented grating stimuli were shown concurrently with the color targets during all phases of experiment 1. In addition, all phases of the experiment were identical with the exception of VTA-EM during the L45° stimulus of the cue-VTA-EM association phase. For clarity all trials depicted show a color target associated with a left hand response although the color target associated with the right hand response occurred equiprobably in all phases.

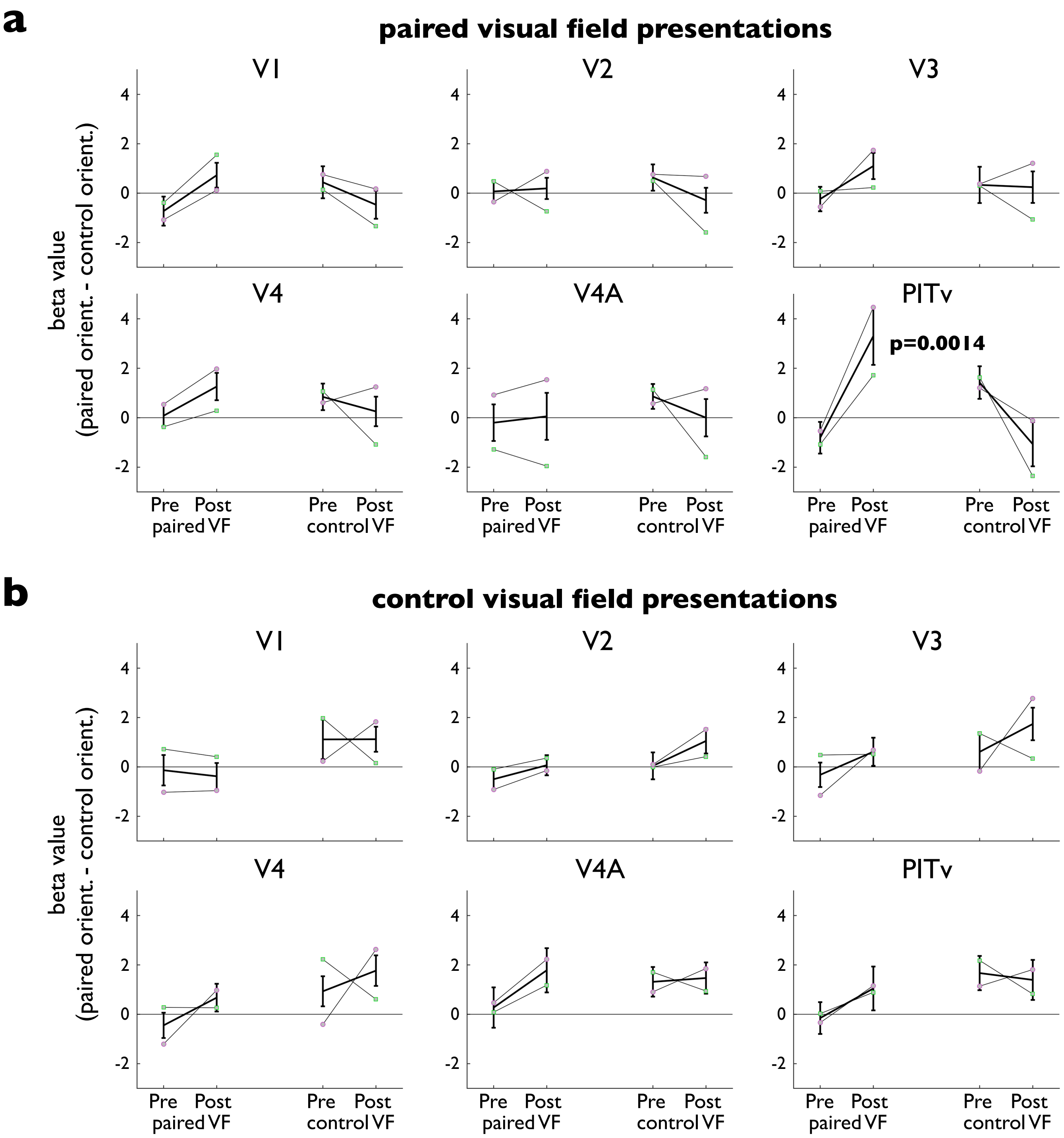

Supplementary Figure 2 | Experiment 1 - fMRI response changes after cue-VTA-EM association. Beta values of the orientation response (trained vs. control orientation) were calculated separately for stimulus presentations in the paired **a** and control visual fields **b** for the pre- (M1 - 104 runs; M2 - 74 runs) and post-association (M1 - 104 runs; M2 - 74 runs) phases of experiment 1. ROIs for the paired [(L45 + L135) vs fixation] and control [(R45 + R135) vs fixation] visual field representations were defined using the combination of an independent localizer experiment (M1 - 65 runs; M2 - 26 runs, P < 0.001) and a probabilistic atlas of retinotopic visual areas (Janssens et al., 2014). Significance of the interaction between pairing and visual field representation was determined using a three-way ANOVA with factors: subject, pairing and visual field. P values were bonferroni corrected for multiple comparisons across 6 ROIs and presentations to both visual fields (paired and control VF). All significant p values are displayed in bold ($p < 0.005$).

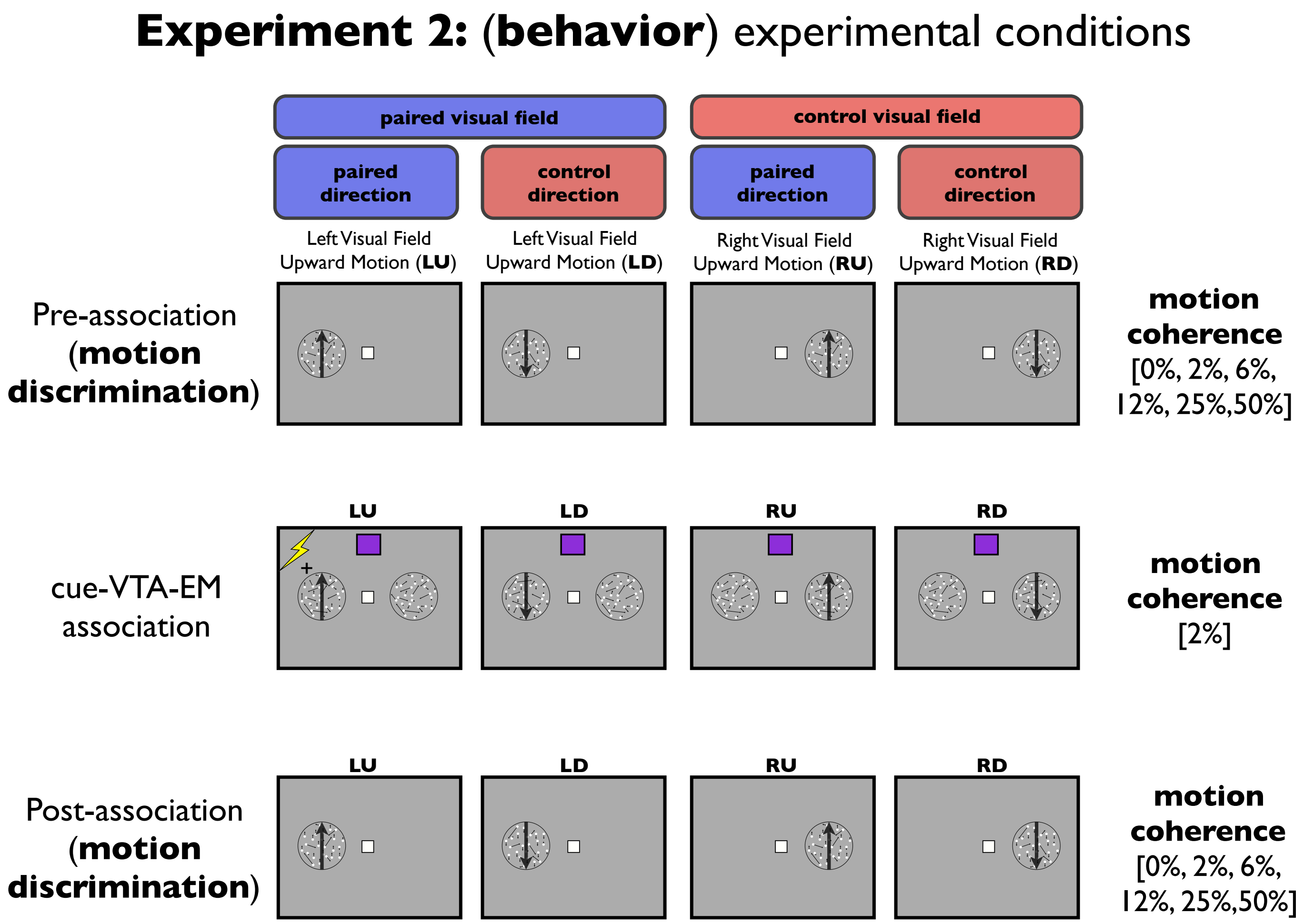

Supplementary Figure 3 | Experiment 2 - behavioral experiment conditions. Schematic of all behavioral experimental conditions used in experiment 2 across the 3 phases of the experiment (pre-association, cue-VTA-EM association, post-association). The schematic depicts one round of experiment 2 in which the upward motion presented in the left VF is paired with VTA-EM during the cue-VTA-EM association phase and is therefore the paired stimulus. The pre- and post-association phases were identical behavioral assessments of the motion discrimination performance. In the pre- and post-association phases both motion directions (upward and downward) were shown in both visual fields (left and right visual fields) and at all motion coherences (0%, 2%, 6%, 12%, 25%, 50%) with equal probability. Upward motion was reported with an upward saccade and downward motion with a downward saccade. During the cue-VTA-EM association the 2% coherent motion stimuli were always shown concurrently with color target stimulus in all conditions. In addition, a 0% coherent motion stimulus was shown before and after the color target presentation in both visual fields. During the 2% motion stimulus a 0% motion stimulus was also displayed in the contralateral visual field. For clarity all of the trials of the cue-VTA-EM association phase depicted show a color target associated with a left hand response although the color target associated with the right hand response occurred with the same frequency.

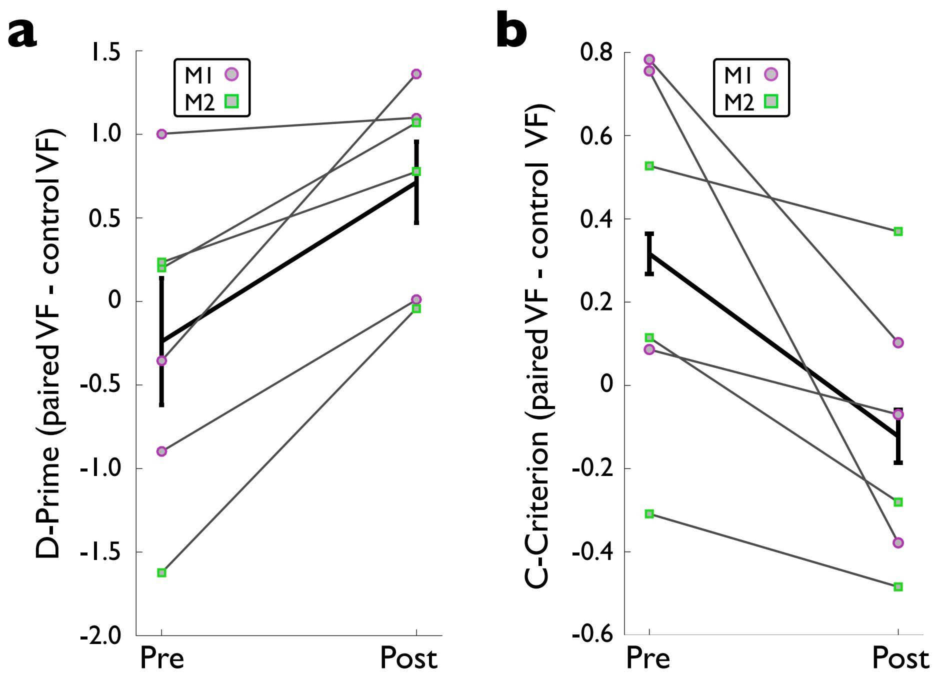

Supplementary Figure 4 | Experiment 2: Effect of cue-VTA-EM association on sensitivity and bias indices for each round of experiment 2. **a** Mean relative motion detection performance (paired VF – control VF) between pre- and post-association phases for parathreshold motion coherence levels (see methods, M1 – 12% coherence, M2 – 6% coherence) is shown for the group (mean across all rounds) and the individuals (each round is shown separately; M1 - purple circle; M2 - green square). Error bars denoting sem for the group data across bins of all rounds. Bins consisted of 100 trials. The pre-association data for each animal in each round (n = 2 monkeys x 3 rounds/monkey) consisted of 40 bins and the post-association data consisted of 20 bins. **(B)** Mean relative motion direction bias (paired VF – control VF) between pre- and post-association phases for all motion coherence levels below 50% (0% - 25%) is shown for the group (mean across all rounds) and the individuals (each round is shown separately; M1 - purple circle; M2 - green square). Error bars denoting sem for the group data across bins of all rounds. Bins consisted of 100 trials. The pre-association data for each animal in each round (n = 2 monkeys x 3 rounds/monkey) consisted of 40 bins and the post-association data consisted of 20 bins. Source data are provided as a Source Data file.

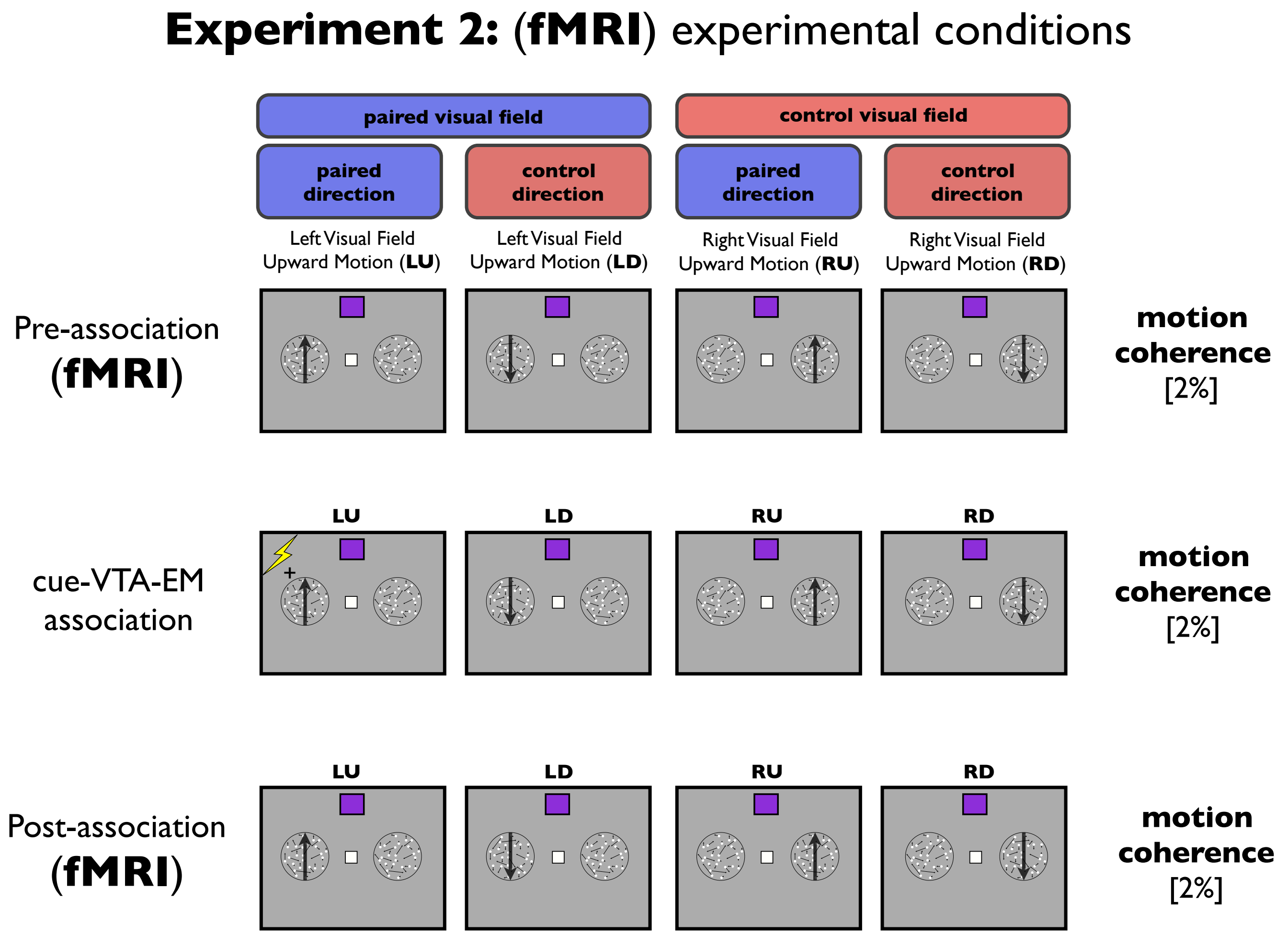

Supplementary Figure 5 | Experiment 3 - fMRI experiment conditions. Schematic of all fMRI experimental conditions used in experiment 2 across the 3 phases of the experiment (pre-association, cue-VTA-EM association, post-association). The schematic depicts one round of experiment 3 in which the upward motion presented in the left VF is paired with VTA-EM during the cue-VTA-EM association phase and is therefore the paired stimulus. The pre- and post-association phases were used to measure fMRI responses to the weak motion stimuli while a concurrent color task was performed. The pre- and post-association phases were identical to the cue-VTA-EM association phase with the addition of the 0% coherence trials and the lack of VTA-EM. During all phases the 2% coherent motion stimuli were always shown concurrently with the color target stimulus. In addition, a 0% coherent motion stimulus was shown before and after the color target presentation in both visual fields. During the 2% motion stimulus a 0% motion stimulus was also displayed in the contralateral visual field. For clarity all of the trials depicted show a color target associated with a left hand response although the color target associated with the right hand response occurred with the same frequency in all phases.

condition (reward probability)	mean baseline MUA (-100 - 0 ms)	mean stimulus response MUA (200-300 ms)	sign-rank (bonferroni corrected p-value)
0%	1.0004	0.9767	5.738×10^{-18}
25%	0.9972	0.9906	5.9×10^{-3}
50%	0.998	1.0338	4.671×10^{-44}
75%	0.9956	1.0582	1.120×10^{-119}
100%	0.9953	1.0762	4.182×10^{-176}

Supplementary Table 1 | Experiment 1 - Positive and negative reward prediction error MUA. Mean baseline (-100 - 0 ms) and stimulus response (200 - 300 ms) MUA activity. To determine significance a sign rank test was performed comparing the baseline and stimulus response. All reward probabilities displayed significant modulation of firing rate ($p < 0.01$; Bonferroni corrected; 0%, n=4823; 25%, n= 4706; 50%, n=4669; 75%, n=4672; 100% n=4668). Source data are provided as a Source Data file.

a

Percent Correct Color Task

	subject	Oriented Grating Stimuli L45°*	L135	R45	R135	
Pre-association	M1	74.03	74.6	73.81	75.07	n = 182 runs, df = 3, Friedman chi-squared = 4.570, p = 0.206
	M2	78.66	81.24	81.21	80.2	

	subject	L45*	L135	R45	R135	
cue-VTA-EM association	M1	75.38	75.96	74.99	75.88	n = 402 runs, df = 3, Friedman chi-squared = 1.922, p = 0.589
	M2	78.25	77.11	77.18	76.8	

	subject	L45*	L135	R45	R135	
Post association	M1	77.5	78.54	76.87	77.02	n = 182 runs, df = 3, Friedman chi-squared = 1.764, p = 0.623
	M2	74.66	75.23	75.77	75.61	

b

Reaction Time (ms) Color Task

	subject	Oriented Grating Stimuli L45*	L135	R45	R135	
pre-association	M1	670.88	668.47	674.35	674.91	n = 182 runs, df = 3, Friedman chi-squared = 0.351, p =0.950
	M2	559.91	559.63	554.46	555.99	

	subject	L45*	L135	R45	R135	
cue-VTA-EM association	M1	584.66	583.95	585.39	579.49	n = 402 runs, df = 3, Friedman chi-squared = 1.221, p = 0.748
	M2	605	592.15	602.64	600.6	

	subject	L45*	L135	R45	R135	
post-association	M1	523.31	527.47	517.64	517.97	n = 182 runs, df = 3, Friedman chi-squared = 2.651, p = 0.448
	M2	597.32	597.33	597.86	601.12	

Supplementary Table 2 | Experiment 1 - Color Task performance. **a** Mean percent correct and **b** mean reaction time on the color task for each of the concurrently displayed low-contrast oriented grating stimuli (L45, L135, R45, R135) during all phases of experiment 1 (pre-association, cue-VTA-EM association, post-association). Significance was determined using a Friedman test. Asterisks denotes the condition paired with VTA-EM. Source data are provided as a Source Data file.

a

Percent Correct Color Task

cue-VTA-EM association	2% motion coherence			
	paired VF		control VF	
subject	paired dir.*	control dir.	paired dir.	control dir.
M1	75.71	75.61	75.87	75.73
M2	81.91	83.53	82.25	82.86

n = 334 runs, df = 3, Friedman chi-squared = 4.052, p = 0.256

b

Reaction Time (ms) Color Task

cue-VTA-EM association	2% motion coherence			
	paired VF		control VF	
subject	paired dir.*	control dir.	paired dir.	control dir.
M1	569.37	568.09	570.37	571.06
M2	672.05	670.5	666.94	669.24

n = 334 runs, df = 3, Friedman chi-squared = 2.288, p = 0.514

Supplementary Table 3 | Experiment 2 - Color Task performance. **a** Mean percent correct and **b** mean reaction time on the color task for each of the concurrently displayed 2% motion condition during the cue-VTA-EM association phase. Significance of the main effect of the motion stimulus was determined using Friedman test. Asterisk denotes the condition paired with VTA-EM. Source data are provided as a Source Data file.

Details of the cue-VTA-EM association phases

subject	experiment	sessions	session length (minutes)	cue-VTA-EM events	cue-VTA-EM events per session
M1	experiment 1 (round 1)	23	173.33	10555	458.91
M2	experiment 1 (round 1)	40	154.06	16648	416.2
M1	experiment 2 (round 1)	19	167.12	9457	497.74
M1	experiment 2 (round 2)	8	178.41	4412	551.50
M1	experiment 2 (round 3)	6	180.82	3242	540.33
M2	experiment 2 (round 1)	7	201.59	3496	499.43
M2	experiment 2 (round 2)	9	218.47	4698	522
M2	experiment 2 (round 3)	20	203.36	9976	498.8

Supplementary Table 4: Number and frequency of cue-VTA-EM events in all rounds of experiment 1 and 2. Details of the cue-VTA-EM association sessions during experiment 1 and experiment 2. subject (left column): identity of subject. Experiment: experiment number and round number. Session: number of cue-VTA-EM association sessions performed. Session Length: mean length of cue-VTA-EM association sessions in minutes. cue-VTA-EM events: total number cue-VTA-EM events. cue-VTA-EM events per session: mean number of cue-VTA-EM events per session.
