# Supplementary material for: Ventral midbrain stimulation induces perceptual learning and cortical plasticity in primates
Source: Nat Commun. 2019 Aug 9;10:3591. doi: 10.1038/s41467-019-11527-9 (PMC6689065; doi:10.1038/s41467-019-11527-9)
Supplement: Supplementary file 3 — Reporting Summary [file 41467_2019_11527_MOESM3_ESM.pdf]

nature research

Corresponding author(s): Wim Vanduffel
John Thomas Arsenault

Last updated by author(s): Jul 8, 2019

Reporting Summary

Nature Research wishes to improve the reproducibility of the work that we publish. This form provides structure for consistency and transparency in reporting. For further information on Nature Research policies, see Authors & Referees and the Editorial Policy Checklist.

Statistics

For all statistical analyses, confirm that the following items are present in the figure legend, table legend, main text, or Methods section.

n/a	Confirmed	
☐	☒	The exact sample size (*n*) for each experimental group/condition, given as a discrete number and unit of measurement
☐	☒	A statement on whether measurements were taken from distinct samples or whether the same sample was measured repeatedly
☐	☒	The statistical test(s) used AND whether they are one- or two-sided<br>*Only common tests should be described solely by name; describe more complex techniques in the Methods section.*
☐	☒	A description of all covariates tested
☐	☒	A description of any assumptions or corrections, such as tests of normality and adjustment for multiple comparisons
☐	☒	A full description of the statistical parameters including central tendency (e.g. means) or other basic estimates (e.g. regression coefficient) AND variation (e.g. standard deviation) or associated estimates of uncertainty (e.g. confidence intervals)
☐	☒	For null hypothesis testing, the test statistic (e.g. *F*, *t*, *r*) with confidence intervals, effect sizes, degrees of freedom and *P* value noted<br>*Give P values as exact values whenever suitable.*
☒	☐	For Bayesian analysis, information on the choice of priors and Markov chain Monte Carlo settings
☐	☒	For hierarchical and complex designs, identification of the appropriate level for tests and full reporting of outcomes
☒	☐	Estimates of effect sizes (e.g. Cohen's *d*, Pearson's *r*), indicating how they were calculated

Our web collection on statistics for biologists contains articles on many of the points above.

Software and code

Policy information about availability of computer code

Data collection	Siemens syngo MR B17
Data analysis	Matlab 2016, Python 3.6.7, SPM 5, R 3.5.1

For manuscripts utilizing custom algorithms or software that are central to the research but not yet described in published literature, software must be made available to editors/reviewers. We strongly encourage code deposition in a community repository (e.g. GitHub). See the Nature Research guidelines for submitting code & software for further information.

Data

Policy information about availability of data

All manuscripts must include a data availability statement. This statement should provide the following information, where applicable:

- Accession codes, unique identifiers, or web links for publicly available datasets
- A list of figures that have associated raw data
- A description of any restrictions on data availability

The data reported in this paper are tabulated in the Supplementary Materials and will be available upon reasonable request. In addition, the source data underlying Figs 1a-b, 4f-g, Supplementary Figs 4a-b and Supplementary Tables 1, 2a-b and 3a-b are provided with the paper as a Source Data file.

Field-specific reporting

Please select the one below that is the best fit for your research. If you are not sure, read the appropriate sections before making your selection.

☒ Life sciences ☐ Behavioural & social sciences ☐ Ecological, evolutionary & environmental sciences

Life sciences study design

All studies must disclose on these points even when the disclosure is negative.

Sample size	Due to ethical imperatives to limit the number of non-human primates used during a study, we restricted these experiments to 2 animals. Because the results were replicated in both animals, we feel this sample size balances reducing the number of animals while still demonstrating the consistency of the observed results. Moreover, this sample size is consistent with the majority of non-human primate experiments.
Data exclusions	No data exclusion was used.
Replication	All the reported results in experiment 1, 2 and 3 were consistent across the two animals. In addition, the behavioral results were consistent across all 3 rounds of experiment 2 in both animals.
Randomization	Individual trial types (grating or motion stimuli) in the pre-association, cue-vta-em association and post-association phases of experiment 1, 2 and 3 were randomly intermixed. Both subjects completed all experiments and therefore were not randomly assigned. Importantly control conditions were always compared to conditions of interest within the same subject to control for intersubject and temporal differences.
Blinding	Blinding was not used but analysis was restricted to quantitative measures.

Reporting for specific materials, systems and methods

We require information from authors about some types of materials, experimental systems and methods used in many studies. Here, indicate whether each material, system or method listed is relevant to your study. If you are not sure if a list item applies to your research, read the appropriate section before selecting a response.

Materials & experimental systems

n/a	Involved in the study	
☒	☐	Antibodies
☒	☐	Eukaryotic cell lines
☒	☐	Palaeontology
☐	☒	Animals and other organisms
☒	☐	Human research participants
☒	☐	Clinical data

Methods

n/a	Involved in the study	
☒	☐	ChIP-seq
☒	☐	Flow cytometry
☐	☒	MRI-based neuroimaging

Animals and other organisms

Policy information about studies involving animals; ARRIVE guidelines recommended for reporting animal research

Laboratory animals	Two rhesus monkeys (Macaca mulatta; M1, M2; 5-7 kg, 4-6 years old, 2 males) were used in this study.
Wild animals	The study did not involve wild animals.
Field-collected samples	The study did not involve samples collected from the field.
Ethics oversight	Animal care and experimental procedures were performed in accordance with the National Institute of Health's Guide for the Care and Use of Laboratory Animal, the European legislation (Directive 2010/63/EU) and were approved by the Animal Ethics Committee of KU Leuven.

Note that full information on the approval of the study protocol must also be provided in the manuscript.

Magnetic resonance imaging

Experimental design

Design type	Monkeys performed a color discrimination task in the scanner during experiment 1 and 3. The design was even-related with different grating or motion stimuli being randomly presented while the animals performed a concurrent color discrimination task.
Design specifications	Experiment 1 fMRI: M1 performed 104 runs and M2 performed 74 runs during the pre-association phase. M1 performed 104 runs and M2 performed 74 runs during the post-association phase. Each run lasted for 600 s. The stimulus presentation/color discrimination duration was 500 ms. The time between trials was 1500 - 2000 ms. Experiment 3 fMRI: M1 performed 77 runs and M2 performed 48 runs during the pre-association phase. M1 performed

	47 runs and M2 performed 43 runs during the post-association phase. Each run lasted for 600 s. The stimulus presentation/color discrimination duration was 500 ms. The time between trials was 1500 - 2000 ms.
Behavioral performance measures	Percent correct and reaction time at the color discrimination task were both tested using an ANOVA across fMRI runs to test for differences between the different grating stimuli (L45°, L135°, R45°, R135°).

Acquisition

Imaging type(s)	functional imaging
Field strength	3 Tesla
Sequence & imaging parameters	gradient echo sequence, EPI, field of view = 105 mm x 105 mm, 84 x 84 matrix size, slice thickness = 1.25 mm, orientation = sphinx, TR=2 s / TE=17 ms / flip angle= 84°.
Area of acquisition	whole brain scan

Diffusion MRI ☐ Used ☒ Not used

Preprocessing

Preprocessing software	SPM 5, JIP 3.1, MATLAB 2016, slice by slice realignment (Kolster, 2009)
Normalization	After images were motion corrected they were non-rigidly normalized using JIP fMRI Analysis Toolkit.
Normalization template	Images were normalized to a template anatomical volume of subject M2 and resampled to 1 mm^3.
Noise and artifact removal	The 6 motion regressors from SPM realignment procedure were used in the GLM mass univariate analysis as a regressor of no interest.
Volume censoring	Experiment 1 mass univariate: Analysis was restricted to an anatomical brain mask of M2.<br>Experiment 1 roi: Analysis was restricted to visual areas (V1 to to inferotemporal cortex) based upon the probabilistic atlas (Janssens, 2014).<br>Experiment 1 searchlight multivariate: Analysis was restricted to visual areas (V1 to to inferotemporal cortex) based upon the probabilistic atlas (Janssens, 2014).<br>Experiment 3 mass univariate: Analysis was restricted to an anatomical brain mask of M2.

Statistical modeling & inference

Model type and settings	Experiment 1 mass univariate: A first-level fixed-effect analysis was performed on each run of each phase performed by each monkey. A t-test was then done comparing the fMRI responses from the post-association phase to the pre-association phase. This was performed separately for each monkey. A conjunction analysis across animals was then performed on the resultant images.<br>Experiment 1 ROI: An ROI analysis was perfomed on all visual areas (V1 to to inferotemporal cortex) based upon the probabilistic atlas (Janssens, 2014). An ANOVA analysis of the orientation response (paired vs. control orientation) was performed with factors phase (pre-association or post-association) x VF (paired VF or control VF) and monkey (M1 or M2) was used to control for effect of monkey.<br>Experiment 1 searchlight multivariate: A naive Bayes classifier using data from a sphere of 20 voxels was used to identify whether fMRI responses were at 45 or 135. This was performed separately for each phase, each visual field and each animal. Leave one out cross validation was used to assess mean accuracy.<br>Experiment 3 mass univariate: A first-level fixed-effect analysis was performed on each run of each phase performed by each monkey. A second level ANOVA analysis was then performed with factors: phase (pre- or post-association) x motion direction (paired vs. control direction) and monkey (M1 or M2) was used to control for effect of monkey.
Effect(s) tested	Experiment 1 mass univariate: The contrasts compared in the second level t-test (post- vs. pre-association) were (R45° vs. R135°) and (L45° vs. L135°).<br>Experiment 1 searchlight multivariate: 4 different effects (L45° vs. fixation, L135° vs. fixation, R45° vs. fixation, R135° vs. fixation) were tested for each of the 2 phases of the experiment (pre-association and post-association) for a total of 8 effects tested.<br>Experiment 1 ROI: From the ANOVA the interaction of phase and VF was tested for each visual region.<br>Experiment 3 mass univariate: The interaction of phase (pre- or post-association) x motion direction (paired vs. control direction) was tested for stimuli presented to the paired vf and the control vf.

Specify type of analysis: ☐ Whole brain ☐ ROI-based ☒ Both

Anatomical location(s)	Experiment 1 mass univariate: Whole brain.<br>Experiment 1 ROI: Analysis was performed separately on all visual areas (V1 to inferotemporal cortex) based upon the probabilistic atlas (Janssens, 2014)<br>Experiment 1 searchlight multivariate: Analysis was restricted to visual areas (V1 to inferotemporal cortex) based upon the probabilistic atlas (Janssens, 2014).<br>Experiment 3 mass univariate: Whole brain.

Statistic type for inference
(See Eklund et al. 2016)

Voxel-wise statistics were reported for the mass univariate analyses of experiment 1 and 3. For the seachlight multivariate analysis p-values were calculated based on a permutation analysis of the probability of conjunction results with p <= 0.005 and cluster size >= 25 voxels.

Correction

For the seachlight multivariate analysis p-values were calculated based on a permutation analysis of the probability of conjunction results with p <= 0.005 and cluster size >= 25 voxels.

Models & analysis

n/a	Involved in the study
☒	☐ Functional and/or effective connectivity
☒	☐ Graph analysis
☐	☒ Multivariate modeling or predictive analysis

Multivariate modeling and predictive analysis

Experiment 1 searchlight multivariate: Independent variables were comprised of a sphere of 20 voxels with data points comprised of per run beta values of contrasts for either left visual field stimuli (L45° vs. fixation and L135° vs. fixation) or right visual field stimuli (R45° vs. fixation and R135° vs. fixation) from either the pre- or post-association phases. The naive bayes classifier from the CoSMoMVPA package was used. Leave-one out cross-validation was used to assess classification accuracy. A conjunction analysis was performed across animals. P-values were calculated based on a permutation analysis of the probability of conjunction results with p <= 0.005 and cluster size >= 25 voxels.
